# The role of exon 45 and 16 in the pathogenesis of Von Willebrand disease in Iranian Patients

**Published:** 2012-09-22

**Authors:** M Nasiri, H Galehdari, M Darbouy, M Yavarian, B Keikhaee

**Affiliations:** 1 Department of Genetics, Science and Research Branch, Islamic Azad University, Fars, Iran.; 2 Research Centre for Thalassemia and Hemoglobinopathies of Ahvaz Jundishapur University of Medical Sciences Ahvaz, Iran.; 3 Hematology Research Centre, Shiraz University of Medical Science, Shiraz, Iran.

**Keywords:** Von Willebrand disease, Von Willebrand Factor, Single Nucleotide Polymorphism (SNP), Mutation, PCR-Sequencing

## Abstract

**Background:**

Von Willebrand disease (VWD) is an autosomal recessive congenital bleeding disorder with deficiency or dysfunction of von Willebrand factor (VWF). The gene encoding for the VWF is located on chromosome 12, which is 178 Kb with 52 exons. Various mutations of this gene is responsible for the clinical features of VWD, but some single nucleotide polymorphisms make the molecular diagnosis of it very complicated.In this study genetic variations in two exons (45 & 16) of VWF gene in Iranian patients suffer from type 3 VWD from south west of Iran were evaluated.

**Materials and Methods:**

Genetic variations in exon 45 and exon 16 of VWF gene were evaluated in 33 patients diagnosed with type 3 VWD from south west of Iran. Two exons with their flanking introns were amplified by PCR and amplicons were analyzed by sequencing for any molecular changes.

**Results:**

No mutation was found in both selected regions. An A/C polymorphism in intron 44 was recognized in all patients in homozygous manner. This SNP reported for the first time from Iranian VWD patients.

**Conclusion:**

Mutation of VWF gene is different in various ethnic groups, which finding of is important in the diagnosis of the VWD, especially for prenatal diagnosis.

A few mutations are reported for exon 45 and 16 of this gene in Iran and other countries. But, present study didn't find any mutation in these patients. Mutation in other exons or introns should be evaluated in affected individuals from south west of Iran.

## Introduction

Von Willebrand disease (VWD) is the most common type of bleeding disorder, affecting up to 1% of the world population (1, 2). The most frequent symptoms experienced by patients are Mucocutaneous bleeding including epistaxis, menorrhagia, gingival bleeding and prolonged bleeding after trauma and surgery (3).

All types of VWD have reduced amounts or abnormal forms of VWF in the circulation. VWF works in hemostasis, which binds to platelets and the subendothelium to promote platelet adhesion. It binds to activated platelets to promote platelet aggregation and finally binds to FVIII to prevent premature degradation of this coagulation cofactor (4). 

VWF is encoded by a gene on human chromosome 12 at 12p13.3. The gene is 178 Kb with 52 exons. VWF is highly polymorphic gene, and up to now many polymorphic variations in exons and closely flanking introns was reported on International Society on Thrombosis and Haemostasis (ISTH). This highly polymorphic gene could complicated molecular analysis and thier result interpretation (1, 5, 6). VWF gives rise to a 9 kb mRNA that encodes a 2,813 amino acid protein which is comprised of a series of domains (7- 9). Each domain is encoded by a cluster of exons. Since, the frequency of mutations in exon 45 and 16 of the VWF gene is very high, these regions are selected as a first choice of mutation screening in our comprehensive project on molecular study of VWD in southwest Iran.

Exon 45 contributes to the synthesis of ‍‍‍C_1_ and C_2_ domains. The Arg-Gly-Asp-Ser (RGDS) sequence at amino acids 1744 to 1747 of the mature VWF subunit among these domains serves as the binding site for GpIIb-IIIa. This complex is a member of integrin family of cell surface receptors. Following platelet activation, GpIIb-IIIa undergoes a conformational change to high affinity ligand binding state (10, 11).

 Exon 16 is responsible for coding the D_2_ domain of propeptide segment of VWF protein precursor in the group of exons include exon 11-17. Propeptide is composed of 741-N terminal amino acids of VWF, which cleaved off by RACE cleaving enzyme at trans Golgi network (6). 

To date, many different mutations are recognized in the VWF gene, and most of them are associated with qualitative defects in specific VWF domains (12). Variety of gene defects lead to the lack of VWF mRNA expression such as nonsense mutations, splice-site mutations and deletions. Missense mutations are not common in type 3 VWD (13). 

Based on ISTH published in 2006, VWD is considered as either a quantitative (type 1 & 3) or qualitative (type 2) trait (14). The uncommon type 3 variant is the most severe form of the disease, and it is characterized by very low or undetectable levels of VWF. They suffer from severe bleeding diathesis, which has generally autosomal recessive pattern of inheritance (15- 17). Type 1, the most common variant, is characterized by normal structure and function of the VWF, but decreased quantity (18, 19). In type 2 VWD, the VWF is abnormal in structure and/or function. Type 2 is divided into four subtypes (2A, 2B, 2M and 2N) with determined phenotypic features. The vertical transmission of type 1 & type 2 VWD suggests the autosomal dominant pattern of inheritance (20).

The role of exon 45 and 16 in VWF function reported in previous study, especially from Iran. So these exons were chosen for mutation screening in the present study. 

## Materials and Methods

Thirty three patients diagnosed as VWD type 3 (11 males and 22 females) were enrolled in this study. Fifty one point five percent (17/33) of patients were born from consanguineous marriages and 54.5% (18/33) of them have Arabian background (the prominent ethnic population in this region of Iran). Seven families have two affected children, and other families have only one child suffer from the disease. 

Laboratory assays for all patients resulted in virtually absent to very low levels of VWF antigen (VWF-Ag <1%). All patients had repeatedly bleeding episodes per year and were treated with FVIII/VWF concentrates.

5 ml whole blood was collected from 33 unrelated patients and their family members after signing informed consent form. In this study, PCR- direct sequencing was used to detect mutations in VWD patients. The PCR primers were designed by online version of the primer 3 software from the flanking intronic sequences at both 5' and 3' end of the exon 45 and 16 (Table 1). Genomic DNA was extracted using AccuPrep Genomic DNA extraction kit (Bioneer Co. South Korea). Based on literature review and the impact of the exon 45 and 16 on VWF function, we selectively searched for disease causing mutations in this exon as a part of our comprehensive project on VWD. Exon 45 and 16 with their flanking intron region were amplified by PCR that was done in a total volume of 20µl containing 10 pmol of each primer (TAG Copenhagen A/S, Fruebjergvej3, and Denmark) and 50ng gDNA. PCR condition included the initial denaturation at 95°C for 5 min followed by 30 cycles at 95 C for 30 sec, annealing at 60°C (exon 45), 59°C (exon 16) for 30sec and 72°C for 45sec with a final extension at 72°C for 7 min. PCR products were finally separated on 2% agarose gel. The amplicons were sequenced by direct sequencing method on an automated ABI sequencer (Applied Biosystems, USA). 

## Results

**Figure 1:**
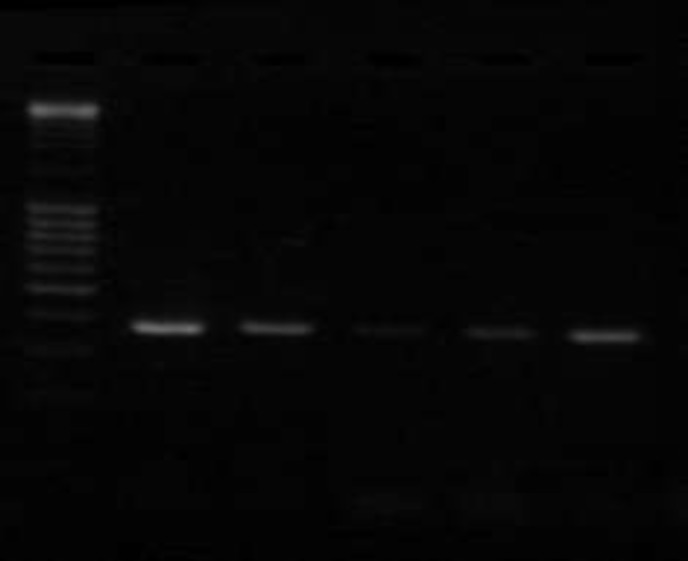
Exon 45 PCR products. the exon and its flanking intronic sequence were amplified by a designed primer set which resulted in 374bp amplicons. 100bp ladder use in the left first lane

**Figure 2:**
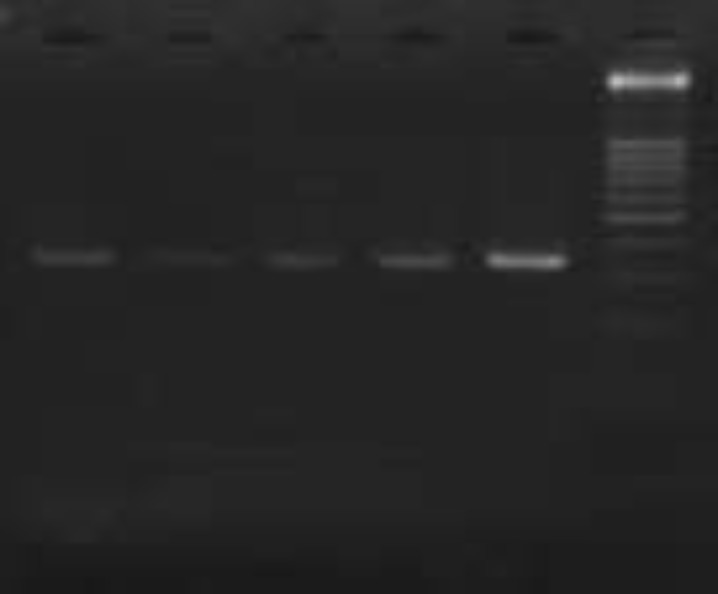
Exon 16 PCR products. the exon and its flanking intronic sequence were amplified by a designed primer set which resulted in 341bp amplicons

**Figure 3:**
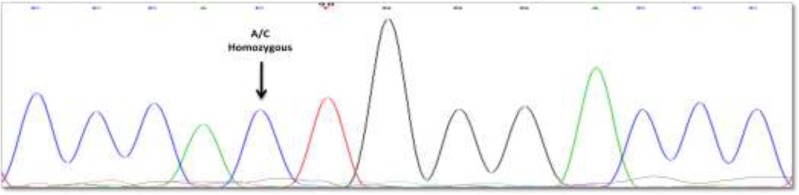
Homozygous pattern for T to C SNP in intron 44 of the VWF gene

Amplification of target sequences using pairs of designed primers for exon 45 and 16 resulted in 374bp and 341bp bands in size respectively (Fig. 1, Fig. 2). Production of only one specific band with the predicted size on the agarose gel emphasize on the accuracy of the PCR amplification. The Sequencing results of amplified fragments by PCR are analyzed by using software Chromas 2.2 and aligning the results of sample sequences with normal sequence on NCBI. No pathogenic mutation was detected in these samples in both exons 45 and 16. One single nucleotide polymorphism (A/C) within intron 44 at position 7549-59 was the only variation in this region of the gene. All patients were homozygous for this polymorphism (Fig. 3). Parents of the patients showed the same SNP in heterozygous manner. Any genetic variation did not find in exon 16 and its intronic sequence at both ends among this population. The effect of the detected SNP (A/C) was evaluated with Fast SNP online software. The analyzing results do not consider any significant role for this SNP on the function and/or structure of VWF.

**Table I:** Summary of designed primers has been used in this study for amplification of exon 45. The primer sets were designed with primer 3 free on line program

**Primer**	**Sequence [from 5’ to 3’]**	**Product length**
**VWF X45-F**	GTGCTCACTGAGACGAGCCCCA	374bp
**VWF X45-R**	CTGCATGCCTTACCACAGCGACAG	
**VWF X16-F**	TCTCTTACCCGGATGAGGAATGCA	341bp
**VWF X16-R**	ACCTGGACCAAATCCCAGCTC	

## Discussion

Different types of pathogenic mutations and a number of single nucleotide polymorphisms (SNPs) were found in the relative large VWF gene (21) so far, which scatter over the entire gene sequence (22, 23). Some gene polymorphisms reported in healthy population. Most SNPs are not responsible for a disease state, but a few might predispose individuals to suffer. So SNPs can be used as biological markers to screen patients for susceptibility to a certain disease by analyzing their DNA for specific SNP profiles (24). Based on literature review, exon 45 and exon 16 select as two of the considerable areas of the gene with large number of mutations. One SNP available in ISTH is also reported repeatedly within intron 44 in some ethnicities. Many researchers document different mutations in exon 45 and exon 16. Baronciani et al report a small insertion (13) and a small deletion (25) resulted in stop codon and as a result the premature and nonfunctional VWF, among Iranian type 3 patients in exon 45. They reported also some small deletions (13) and single nucleotide substitution (25), which result in frame shift and stop codon respectively in exon 16. There are also some reports of missense mutations in EU by Goodeve et al (26). A number of nonsense mutations are from Germany (27), Italy (13) and Sweden (28) that cause VWF reduction as expected in type 3 VWD. Corrales et al also report a dinucleotide (AG) insertion at position7664-7665, which shift the C2557S to terminus (29). Moreover, other di-nucleotide deletion in 2124-2125 position also reported in exon 16 by Gaucher in 1994 in type 2A patients (30). In almost all reports, nonsense mutations change critical amino acids, which cause lack or significant reduction of VW. The presence of those mutations is shown to be compatible with type 3 of VWD. In contrast, the results of present study are not compatible with previous reports, which provide new perspective on this gene for south west Iranian population to investigate molecular pathogenesis of VWD in this area. The A/C polymorphism at nucleotide 7549-59 was found in the present study. This SNP is reported from different countries in Europe and South America, which registered in ISTH but none of them have published yet. This is the first report of mentioned SNP in Iranian population. This SNP introduce a restriction site for restriction enzyme Bsr I in the VWF gene. All the patients in this study were homozygous for that SNP. There is no explanation for this observation. It could be used as a biologic marker for diagnosis the predisposed people for VWD, which should be investigated in a large size study.
